# Association between single nucleotide polymorphisms in oxytocin and vasopressin receptor genes and symptom severity of autism spectrum disorder– preliminary study

**DOI:** 10.1192/j.eurpsy.2021.250

**Published:** 2021-08-13

**Authors:** K. Wilczyński, L. Cichoń, A. Auguściak-Duma, A. Sieroń, M. Janas-Kozik

**Affiliations:** 1 Department Of Psychiatry And Psychotherapy Of Developmental Age, Medical University of Silesia, Katowice, Poland; 2 Department Of Psychiatry And Psychotherapy, John Paul’s II Pediatric Center, Sosnowiec, Poland; 3 Department Of Molecular Biology, Medical University of Silesia, Katowice, Poland, Katowice, Poland

**Keywords:** oxytocin, vasopressin, autism, social

## Abstract

**Introduction:**

One of the defining features of autism spectrum disorder (ASD) are deficits in social interaction and communication. Although their etiology is poorly understood, several lines of evidence from studies on humans and rodents suggest that two nonapeptides – oxytocin and vasopressin – might play a pivotal role in their development.

**Objectives:**

To evaluate if single nucleotide polimorphisms in OXTR and AVPR1A genes are linked to the severity of symptoms in autism spectrum disorder.

**Methods:**

The study was conducted on the group of 40 Caucasian males with average age of 14,22 (SD: 1,71) years. ADOS-2 examination was utilized for confirmation of ASD diagnosis as well as evaluation of symptoms severity in each patient. The genotyping of preselected SNPs for each gene (rs10877969; rs7294536; rs2254298; rs53576) was conducted.

**Results:**

“CC” genotype at rs7294536 (p=0,033) was significantly associated with higher outcomes of ADOS-2 especially in terms of social affect. In case of oxytocin receptor gene, frequency of “AA”/”AG” genotype at rs2254298 equaled 100% and of “AA”/”AG” genotype at rs53576 equaled 85% of the study group (expected “A” allele frequency in neurotypical European population was respectively 11% and 35% according to 1000Genomes database). For rs10877969 prevalence of “CC”/”CT” genotype equaled 95% while expected frequency of “C” allele in neurotypical European population was 13%.

**Conclusions:**

Overrepresentation of minor alleles at rs2254298, rs53576 and rs10877969 in patients with ASD might indicate their link to development of ASD. Furthermore, significant association between minor allele at rs7294536 and symptoms severity suggest potential role of arginine-vasopressin receptor deficiency in clinical picture of ASD.

**Disclosure:**

No significant relationships.

